# Supplementary material for: Cellular basis of accelerated whole-tooth regeneration
Source: bioRxiv. 2026 Jan 7:2026.01.06.697137. Preprint. [Version 1] doi: 10.64898/2026.01.06.697137 (PMC12803150; doi:10.64898/2026.01.06.697137)
Supplement: Supplement 1 [file media-1.pdf]

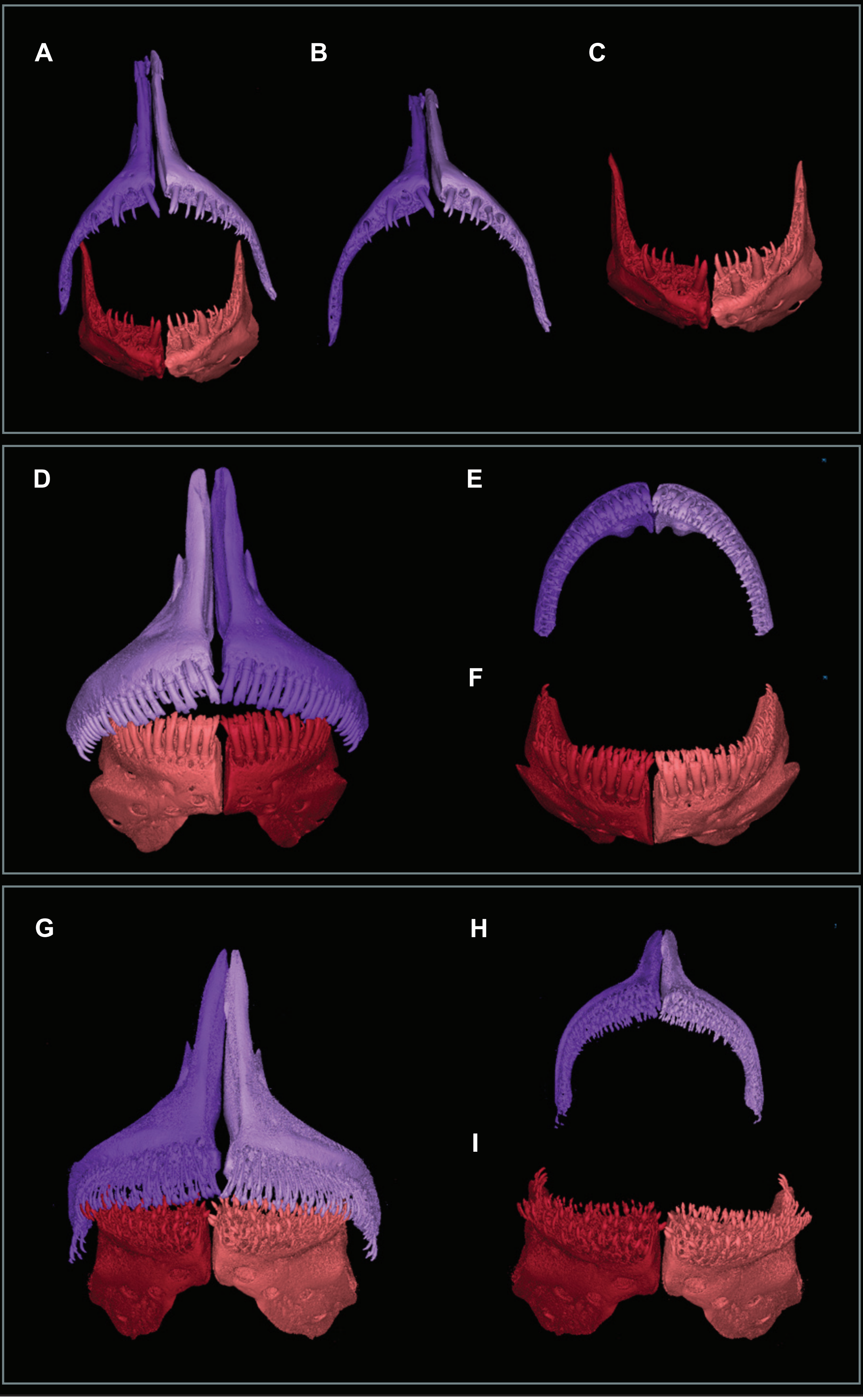

Figure S1 - Micro-CT images of cichlid species exhibiting diverse tooth formulae. (A-C) *Cynotilapia afra* (CA), (D-F) *Metriaclima estherae* (MZ Red), and (G-I) *Petrotilapia chimba* (PT; thick bar). Panels A, D, and G show combined views of the upper and lower jaws; panels B, E, and F show upper Jaws; and panels C, F, I show lower jaws.

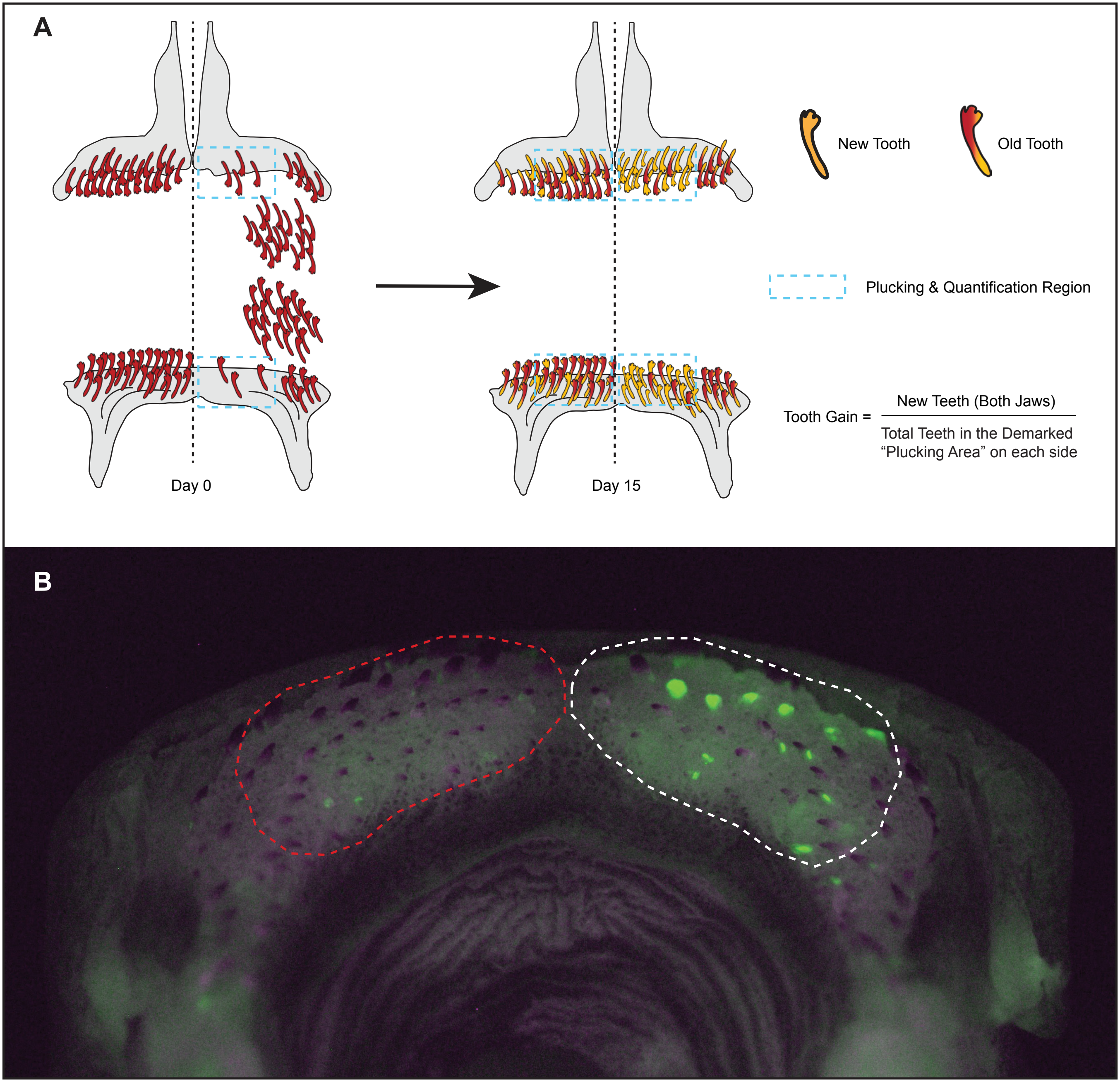

Figure S2 - Schematic overview of tooth-plucking experiments. (A) Following Alizarin Red staining, the majority of teeth on the right side of both the upper and lower jaws were removed. Tooth plucking was restricted to the anterio-lateral region of the jaw, as demarcated by the dotted square. Tooth removal was performed under anesthesia as described in the Methods. (B) Representative lower jaw from *Metriaclima estherae* (MZ Red) 15 days post pluck-control experiment. Dotted outlines indicate regions used for quantification: white denotes the plucked side, and red indicates the unmanipulated control side. These regions correspond to the areas used for quantifying newly formed and old teeth on both sides of the jaw. Whole-jaw imaging was acquired using Zeiss Stereo Discovery.V12. Teeth were individually classified and counted using Zeiss Axio Observer Z1 fluorescence.

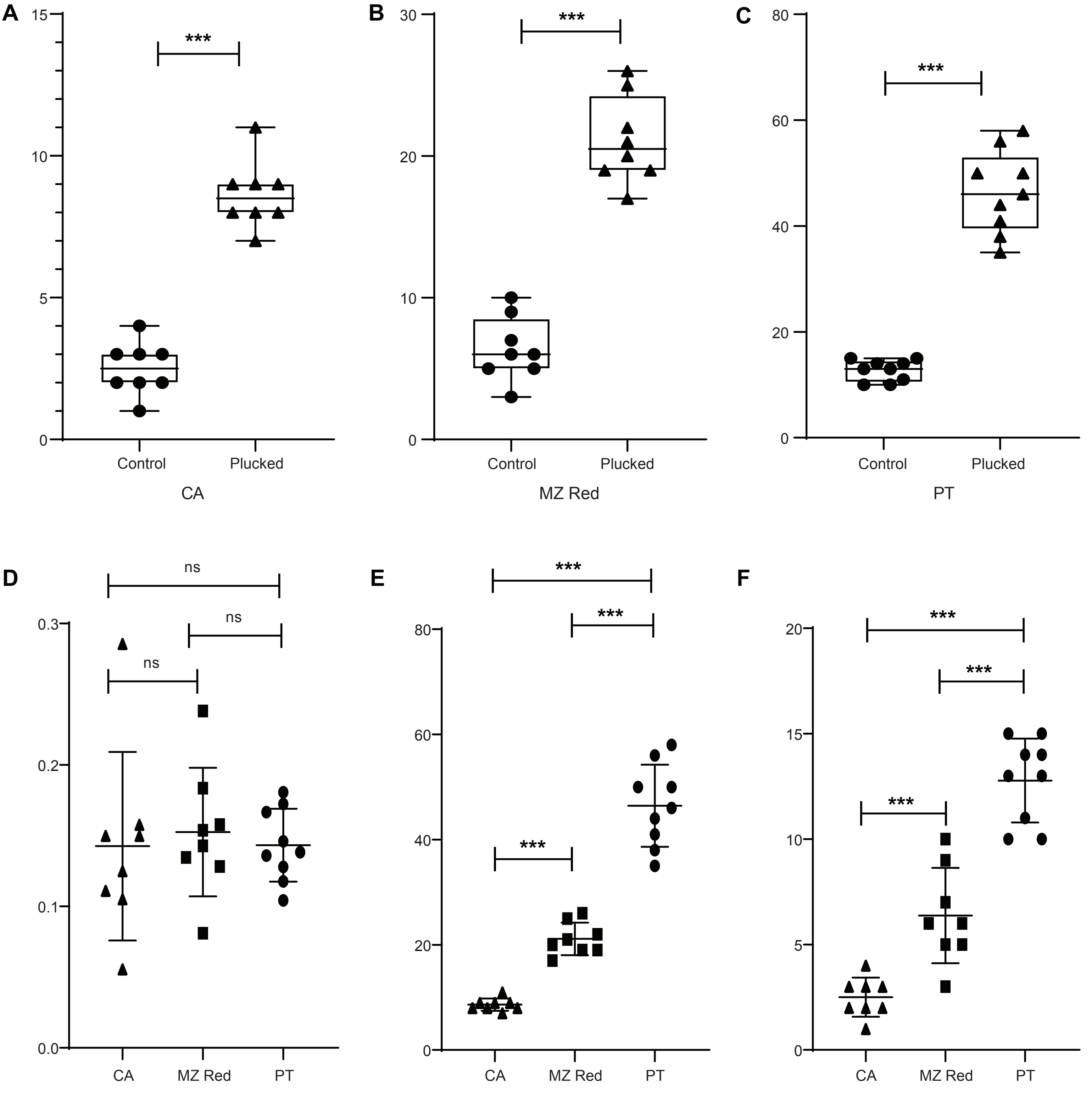

Figure S3 - Pulse-chase reveals elevated rates and number of new tooth formation on the plucked side. (A-C) Number of newly formed teeth in *Cynotilapia afra* (CA), *Metriaclima estherae* (MZ Red), and *Petrotilapia chimba* (PT), respectively. *** denotes *p-value* < 0.0001, paired Student's t-test; sample sizes: CA (n=8), MZ Red (n=8), and PT (n=9). (D) Rate of tooth gain at 15 days was similar across species on the control side. (E) Comparison of the total number of newly formed teeth on plucked side across species. (F) Comparison of total number of newly formed teeth on control side across species. (D-F) *** denotes *p-value* < 0.0001, one-way ANOVA with Tukey-Kramer post-hoc test; sample sizes: PT (n=9), CA (n=8), and MZ Red (n=8).

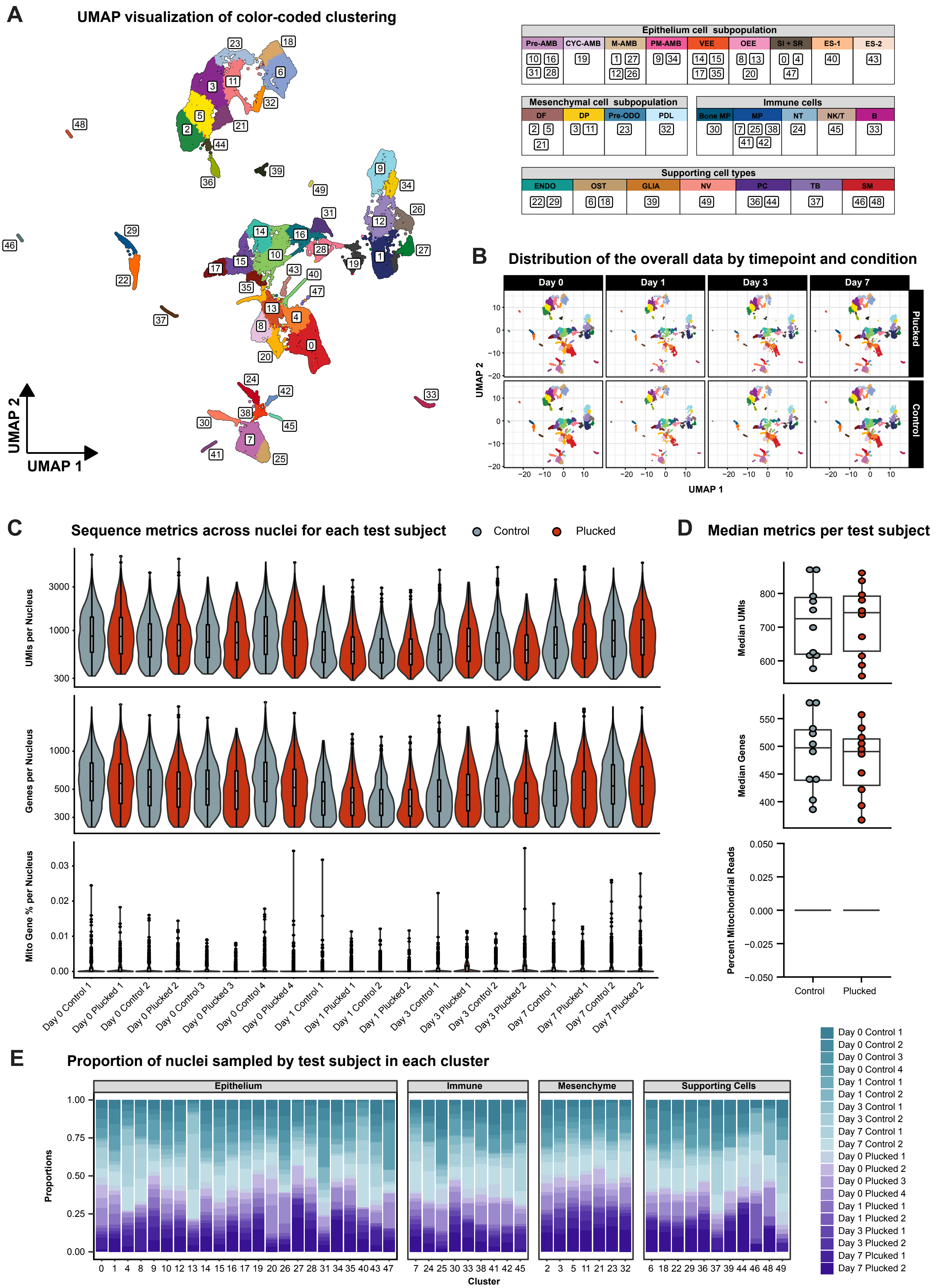

Figure S4 - Single nuclei sampling, library metrics, and cluster composition. (A) Uniform manifold approximation and projection (UMAP) of 27,114 dental cells. Points that are close together represent cells with similar gene expression profiles. The color of each point represents the Seurat clusters (n=50). (B) Cluster composition is consistent across timepoints and between conditions. Individual UMAP plots of nuclei sampled from all 10 test subjects, organized into 4 pairs with plucked subjects shown in the top row and paired control subjects shown in the bottom row. (C) Sequence metrics across nuclei for each test subject (x-axis), including total number of Unique Molecular Identifiers (UMIs, top panel), total number of unique genes (middle panel), and percentage of genes that were mitochondrial (bottom panel). In box plots, the center line indicates the median, the bounds of the box indicate the upper and lower quartiles, and whiskers indicate 1.5x interquartile range. (D) Sequence metrics are consistent between contions and across test subjects. Median sequence metrics across test subjects for each experimental condition (x-axis), including median number of Unique Molecular Identifiers (UMIs, top panel), meidan number of unique genes (middle panel), and median percentage of genes that were mitochondrial (bottom panel). (E) Stacked bar chart showing consistent sampling of clusters across test subjects.

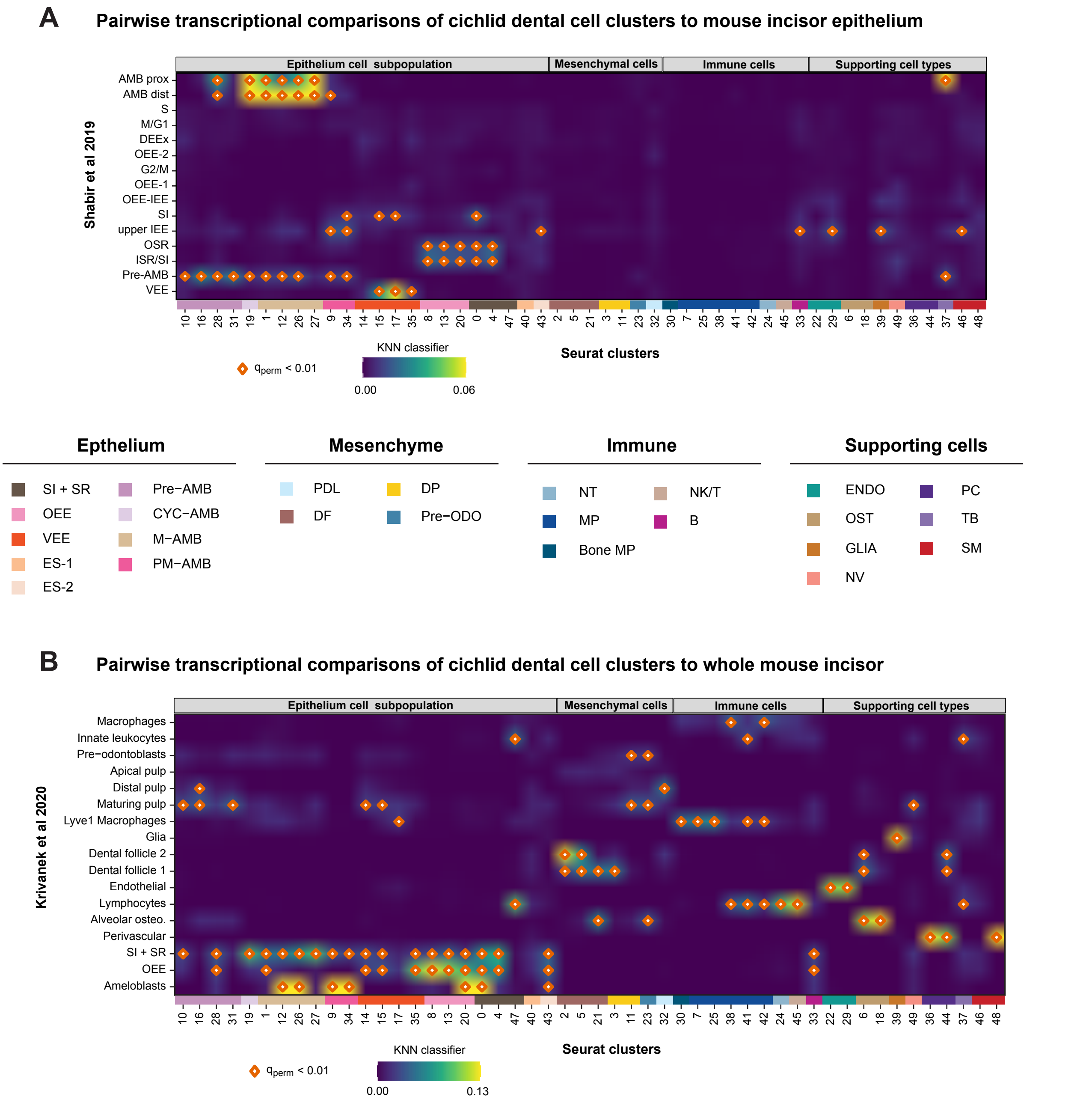

Figure S5 - SAMap-based identification of homologous cell types between cichlid dental tissue and mouse incisor atlases. (A) Similarity scores between cichlid clusters (x-axis) and mouse cell-types (y-axis) in Sharir *et al.*, 2019, which profiled only the proximal region of the mouse incisor epithelium. (B) Similarity scores between cichlid clusters (x-axis) and mouse cell-types (y-axis) in Krivanek *et al.*, 2020, which sequenced the entire mouse incisor, including both epithelial and mesenchymal components. The heatmap scale indicates degree of overlap, with yellow representing higher homology between matched cell types. The dots denote significance determined by permutation testing (qperm<0.01, nperm=1000).

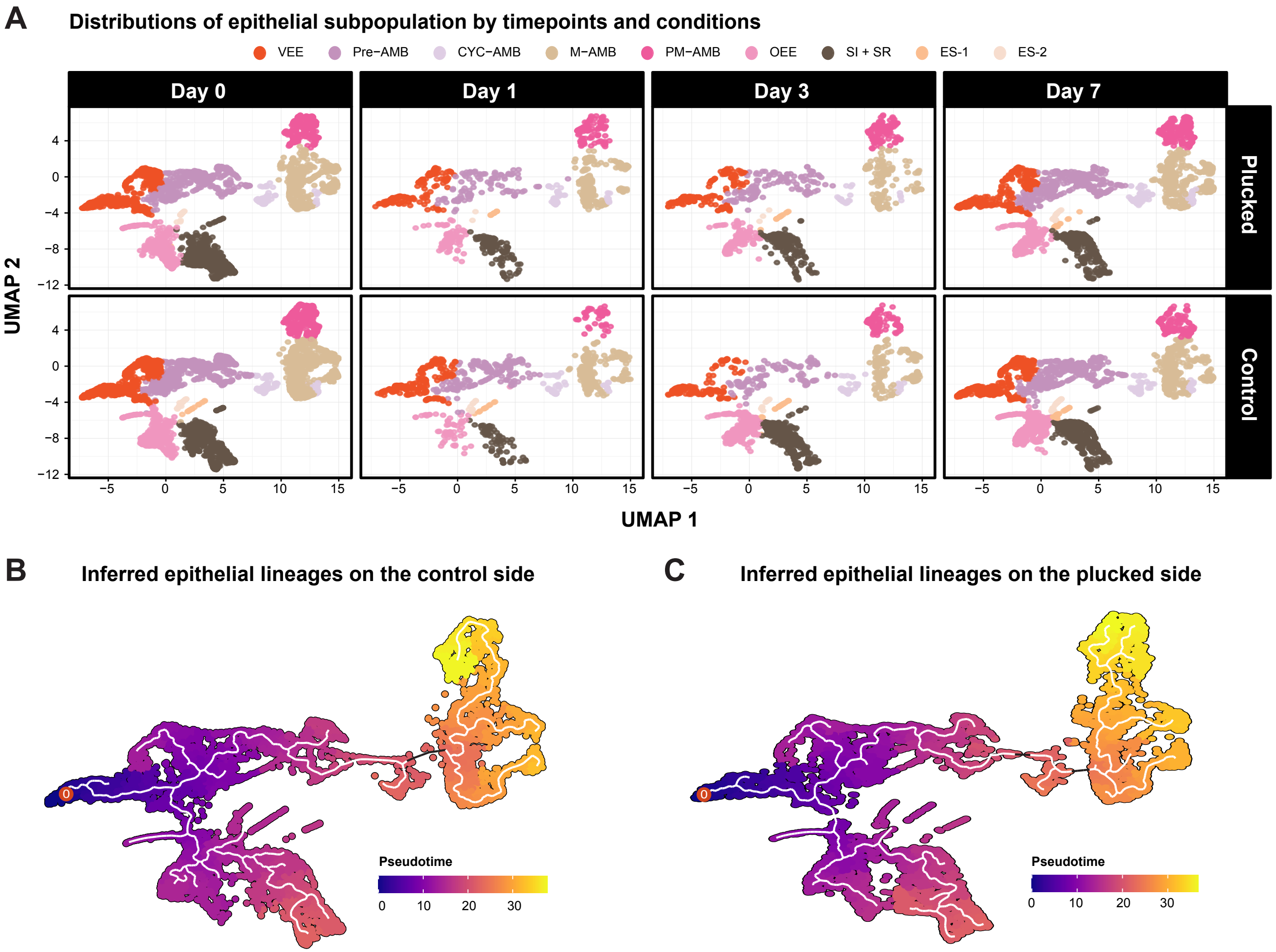

Figure S6 - Developmental trajectory of the epithelial subpopulation. (A) Epithelial subtype composition is consistent across timepoints and between conditions. Individual UMAP plots of nuclei sampled from all 10 test subjects, arranged in four columns by timepoint. Each column represents a matched pair, with the plucked subjects displayed in the top row and the corresponding control subjects in the bottom row. (B-C) UMAP embeddings of control (left) and plucked (right) epithelial cells colored by pseudotime. Cells appearing earliest in pseudotime are denoted by dark purple, and those latest in pseudotime are in yellow.

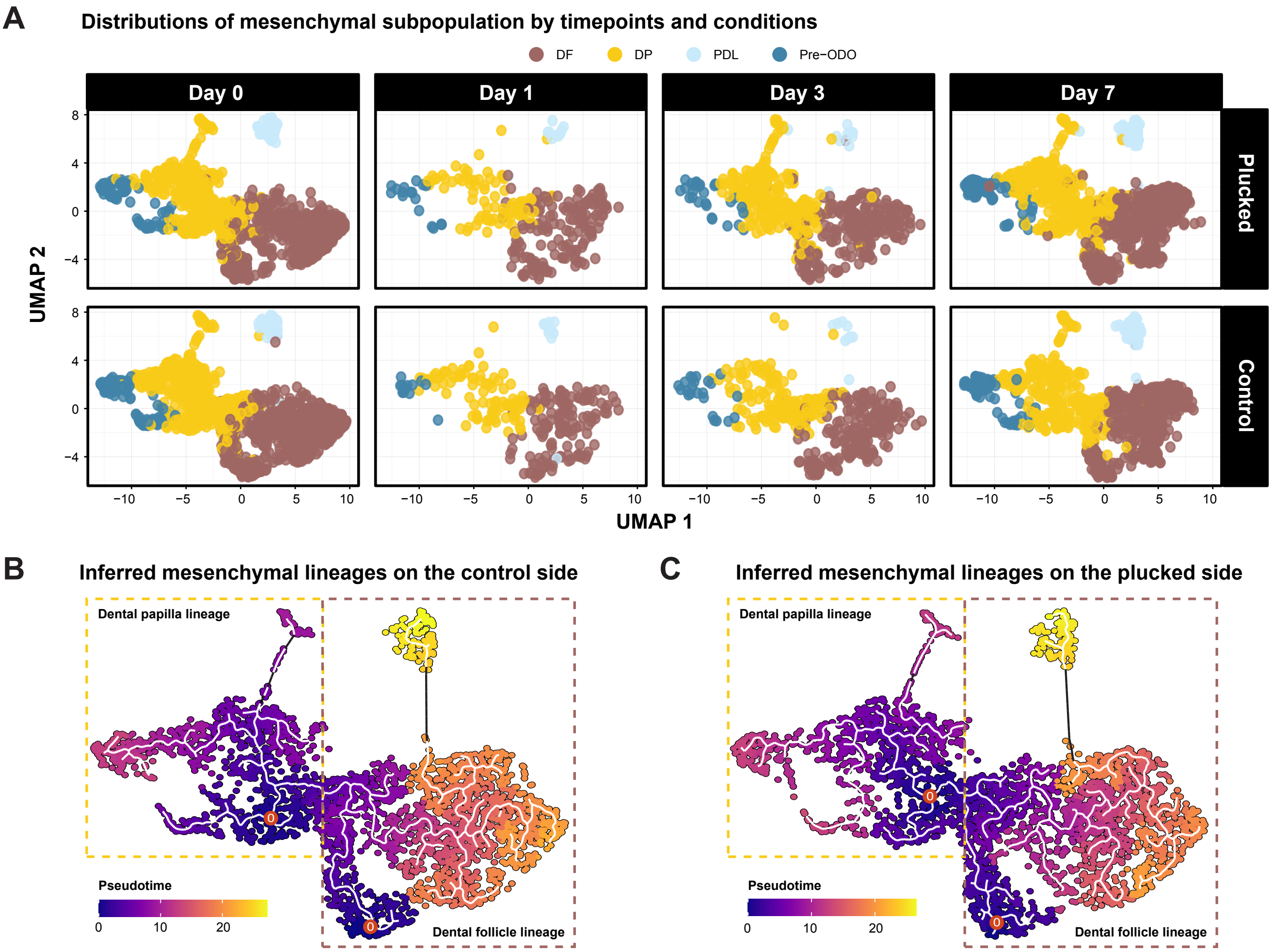

Figure S7 - Developmental trajectory of the mesenchymal subpopulation. (A) Mesenchymal subtype composition is consistent across timepoints and between conditions. Individual UMAP plots of nuclei sampled from all 10 test subjects, arranged in four columns by timepoint. Each column represents a matched pair, with the plucked subjects displayed in the top row and the corresponding control subjects in the bottom row. (B-C) UMAP embeddings of control (left) and plucked (right) mesenchymal cells colored by pseudotime. Cells appearing earliest in pseudotime are denoted by dark purple, and those latest in pseudotime are in yellow.

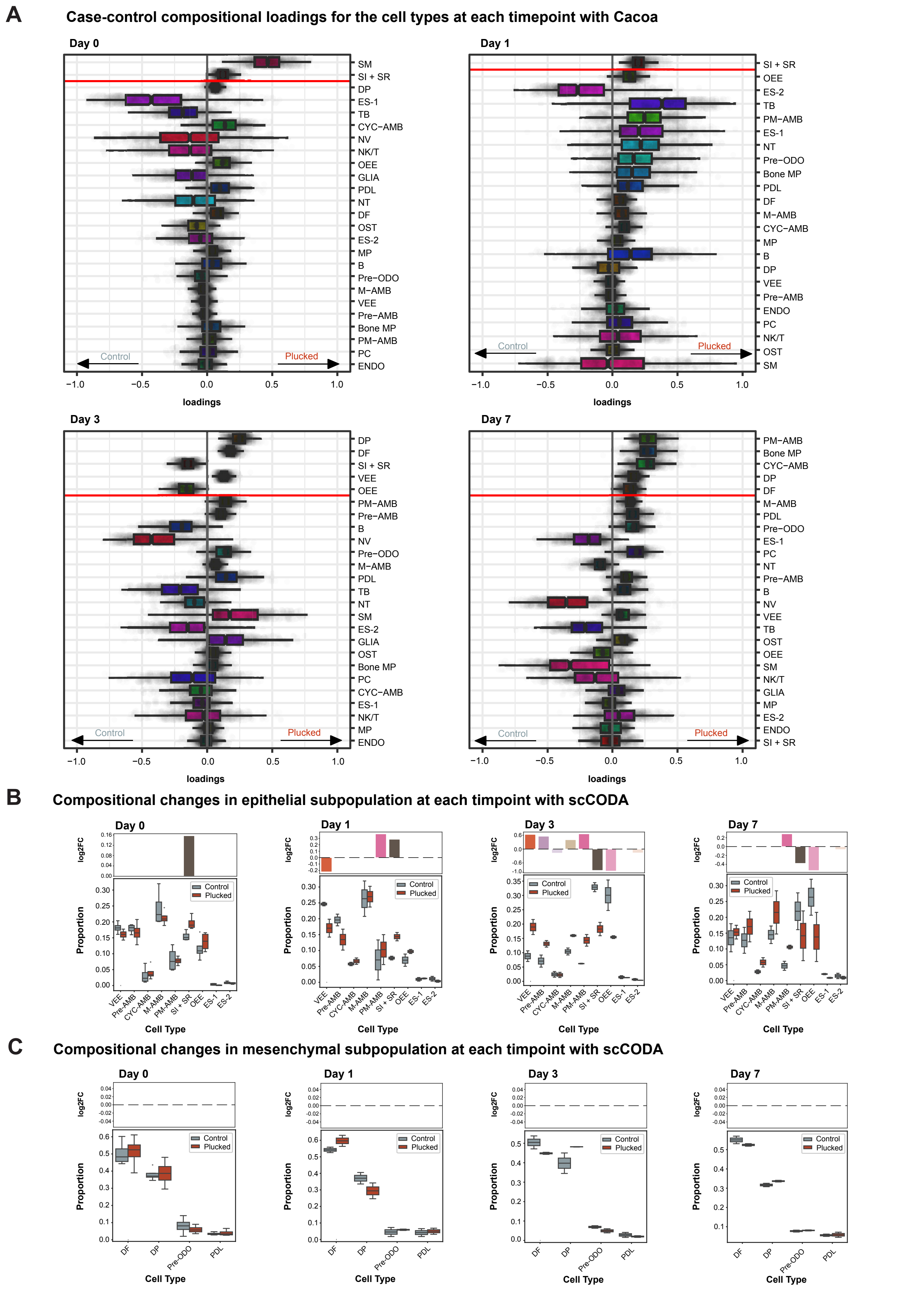

Figure S8 - Compositional analysis of cichlid dental cell types across timepoints using singke-nucleus RNA sequencing. (A) Cacoa case-control compositional loadings (x-axis) for the cell types (y-axis) eact each timepoint. Positive loadings correspond to over-representation in the plucked cells and negative loadings mean over-representation in control cells. The uncertainty of the loading coefficients obtained by resampling with 1000 bootstrapping was represented using boxplot, where the boxes are IQRs split by the median (middle line) and the whiskers represent minimum and maximum loading coefficients. The red horizontal line separates cell types passing significance threshold (*p*-value < 0.05 after Benjamini Hochberg correction). (B-C) The scCODA v.0.1.9 was used for compositional analysis of the epithelial subpopulation (B) and mesenchymal subpopulation (C). The false discovery rate (FDR) value was set to 0.4 to be able to detect subtle yet biologically relevant changes, as described by the authors in their documentation. In all boxplots, the central line denotes the median, boxes represent the IQR, and whiskers show the distribution except for outliers. Outliers are all points outside 1.5× the IQR. In all barplots, a value of zero means that no statistically credible effect was detected. For a value other than zero, a credible change was detected. A positive sign indicates increased abundance in plucked cells, and a negative sign denotes decreased abundance relative to control cells.

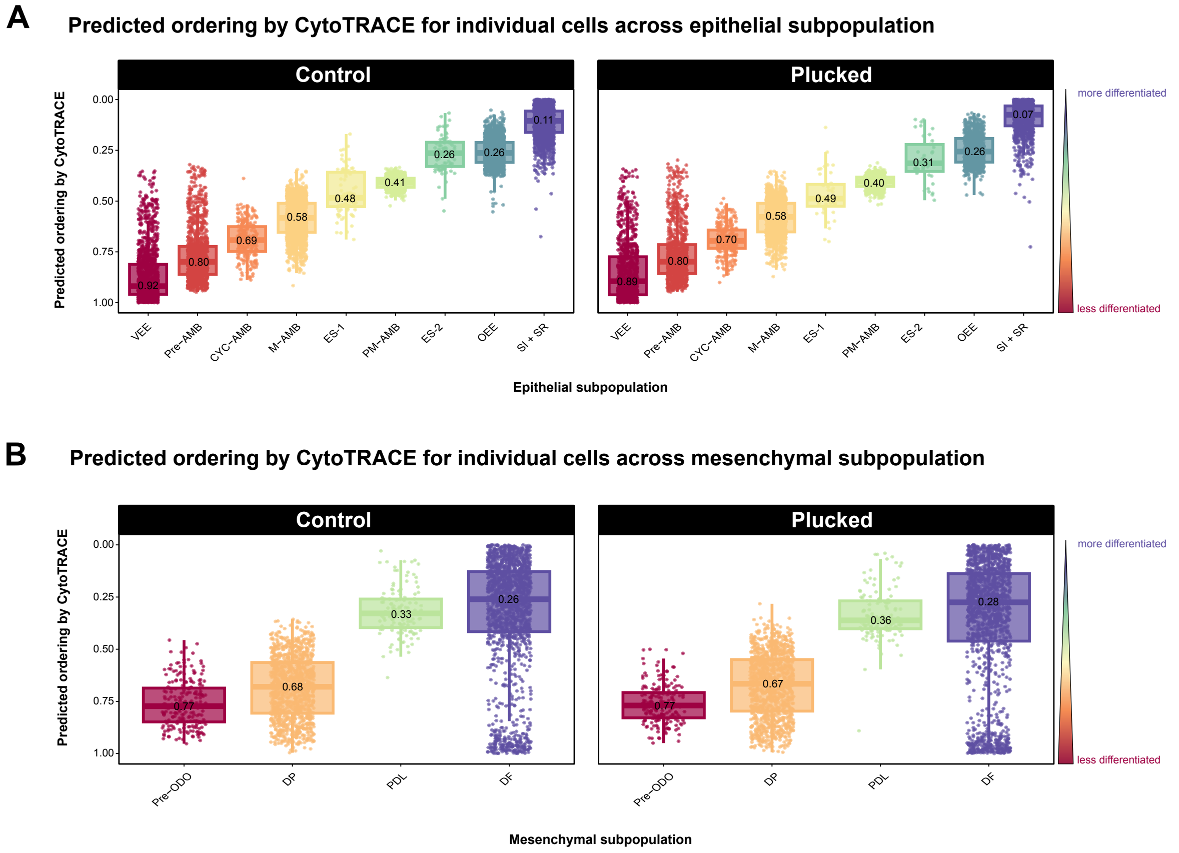

Figure S9 - CytoTRACE-predicted cell state in epithelial and mesenchymal subpopulations. Box-and-whisker plots show the predicted ordering based on CytoTRACE scores for individual cells across (A) epithelial and (B) mesenchymal subpopulations by condition. CytoTRACE scores range from 0 to 1, where higher scores indicate greater developmental potential (less differentiated states) and lower scores indicate more mature, differentiated states.
